# Identification of novel noncoding transcripts in telomerase-negative yeast using RNA-seq

**DOI:** 10.1038/srep19376

**Published:** 2016-01-20

**Authors:** Rachel O. Niederer, Nickolas Papadopoulos, David C. Zappulla

**Affiliations:** 1 Department of Biology, Johns Hopkins University, Baltimore, MD, 21218 USA; 2 Ludwig Center for Cancer Genetics and Therapeutics, The Johns Hopkins Sidney Kimmel Comprehensive Cancer Center, Baltimore, MD 21231 USA

## Abstract

Telomerase is a ribonucleoprotein that maintains the ends of linear chromosomes in most eukaryotes. Loss of telomerase activity results in shortening of telomeric DNA and eventually a specific G2/M cell-cycle arrest known as senescence. In humans, telomere shortening occurs during aging, while inappropriate activation of telomerase is associated with approximately 90% of cancers. Previous studies have identified several classes of noncoding RNAs (ncRNA) also associated with aging-related senescence and cancer, but whether ncRNAs are also involved in short-telomere-induced senescence in yeast is unknown. Here, we report 112 putative novel lncRNAs in the yeast *Saccharomyces cerevisiae*, 41 of which are only expressed in telomerase-negative yeast. Expression of approximately half of the lncRNAs is strongly correlated with that of adjacent genes, suggesting this subset may influence transcription of neighboring genes. Our results reveal a new potential mechanism governing adaptive changes in senescing and post-senescent survivor yeast cells.

Telomerase is a ribonucleoprotein complex that maintains the ends of linear chromosomes, known as telomeres, in most eukaryotes. Telomeres consist of simple, repetitive DNA sequences and a specific set of DNA-binding proteins that protect the ends of chromosomes from erosion or fusion, caused by being processed like double-strand breaks. Thus, telomeres are required for proper maintenance of chromosomes to promote genome stability. In humans, telomerase establishes telomere length early in lifespan, but the enzyme is also aberrantly upregulated in most cancers^1^.

Telomerase consists minimally of a reverse transcriptase, TERT, and a dedicated RNA component, which provides both a flexible scaffold for assembling protein subunits as well as the template for reverse transcription^2^,^3^. In the yeast *Saccharomyces cerevisiae,* cells lacking functional telomerase experience progressive shortening of telomeres and eventually enter a specific cell-cycle arrest in G2/M, known as senescence^4^,^5^,^6^. Later, a subpopulation of cells that maintain their telomeres via recombination-mediated mechanisms, known as survivors, emerge^7^. The changes in gene expression required during these transitions have not been fully elucidated.

Upregulation of telomerase is associated with approximately 90% of cancers and is thought to be required for cells to undergo limitless divisions^1^. In contrast, telomeres have been shown to shorten with aging in mammals as a result of telomerase being largely inactive in somatic cells. Long noncoding RNAs (lncRNAs) have been implicated in both aging and cancer development^8^,^9^ but a specific link between telomerase and changes in lncRNA expression has not been established. Previous work has examined the transcriptional response to both telomerase deletion and telomere decapping using microarray technologies^10^,^11^, however these studies did not include examination of lncRNA expression.

High-throughput technologies have revealed pervasive transcription in *S. cerevisiae*, indicating that approximately 85% of the genome is transcribed^12^,^13^. Among the transcripts are several classes of noncoding RNA (ncRNA), including Xrn1 Unstable Transcripts (XUTs), Cryptic Unstable Transcripts (CUTs), Nrd1-Unterminated Transcripts and Stable Unannotated Transcripts (SUTs) (reviewed in ref. 14). In many cases, the function of these transcripts is unknown, although they can regulate gene expression by influencing histone modifications or by interfering with transcription of nearby genes. There are additional classes of ncRNAs only expressed under specific circumstances, such as meiosis (MUTs)^15^, or in the absence of nonsense-mediated decay (CD-CUTs)^16^, underscoring the potential importance of ncRNAs in regulating adaptive changes in *S. cerevisiae.*

To identify novel noncoding RNAs associated with telomerase-negative cells, we monitored the transcriptome of yeast cells after deleting the telomerase RNA gene. We identified 112 putative new lncRNAs, 41 of which are only upregulated in telomerase-negative cells and that we have termed TMLs (Telomerase-Mutant LncRNAs), whereas the remainder represent novel additional SUTs (Stable Unannotated Transcripts)^17^. Approximately half of the novel lncRNAs show strongly correlated expression with the adjacent genes, suggesting these lncRNAs may influence expression of neighboring genes. Our results indicate that telomerase deletion does induce expression of a specific class of lncRNAs, potentially representing an additional regulatory mechanism in budding yeast.

## Results

### Characterization of the senescence time course of telomerase-mutant yeast cells

**Figure 1:**
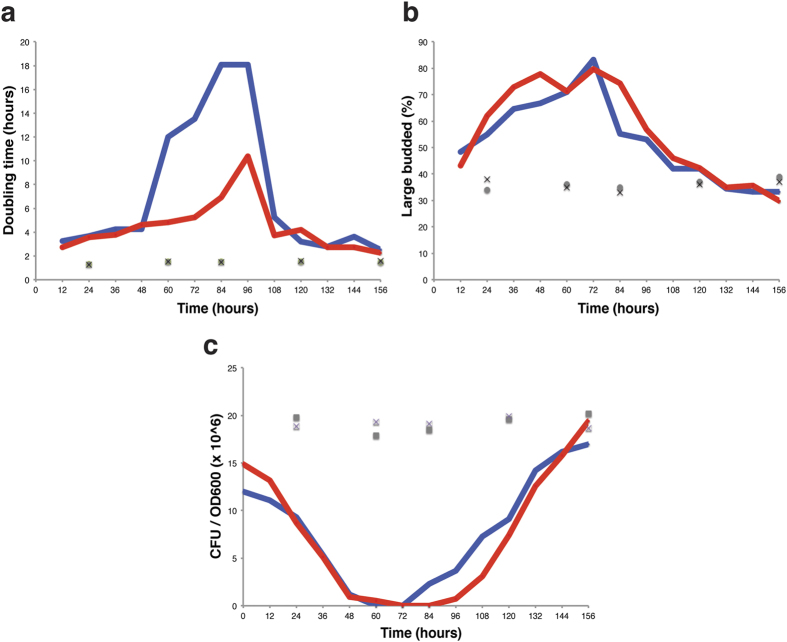
Phenotypic characterization of senescing and post-senescence *tlc1*∆ cells. **(a)** Doubling time between passages. **(b)** Percent G_2_/M cells, monitored by cellular bud indexing. **(c)** Cell viability as measured by ability to form a colony per unit of cell density in culture (optical density based on absorbance of 600-nm light (A_600_)). *tlc1*∆ replicate A shown in red, replicate B shown in blue, nutrient-matched wild-type A and B replicates shown as grey circles or a black ‘x’ respectively.

To identify novel lncRNAs associated with telomerase-negative cells, we generated a strain lacking the telomerase RNA subunit (*tlc1*∆) by sporulation and subsequent dissection of a *TLC1*/*tlc1Δ* heterozygote. Biological-replicate haploids were passaged every 12 hours to maintain logarithmic growth. To identify time points of interest, viability and budding index were examined at each passage. Additionally, DNA and RNA samples were collected to determine telomere length and perform RNA-seq, respectively. Consistent with the onset of senescence, cellular division potential decreased while the percent of G2/M-arrested cells increased early in the time course, reaching a maximum between 2–3 days after the start of culturing (Fig. 1). After this point, both viability and doubling time began to return to wild-type levels, concurrent with the appearance of survivors. Analysis of the telomere Southern blot confirms the formation of type-II survivors, which is expected in liquid culture^7^,^18^ (Supp. Fig. 1). Based on the phenotypic characterization, 5 time points were chosen for further analysis by RNA-seq corresponding to the following stages: (1) presenescence, (2) early senescence, (3) late senescence, (4) early survivor and (5) survivor. Nutrient-matched wild-type *TLC1* cells were generated as controls to identify changes in transcription specific to telomerase-negative cells.

### Identification of putative noncoding RNAs

**Figure 2:**
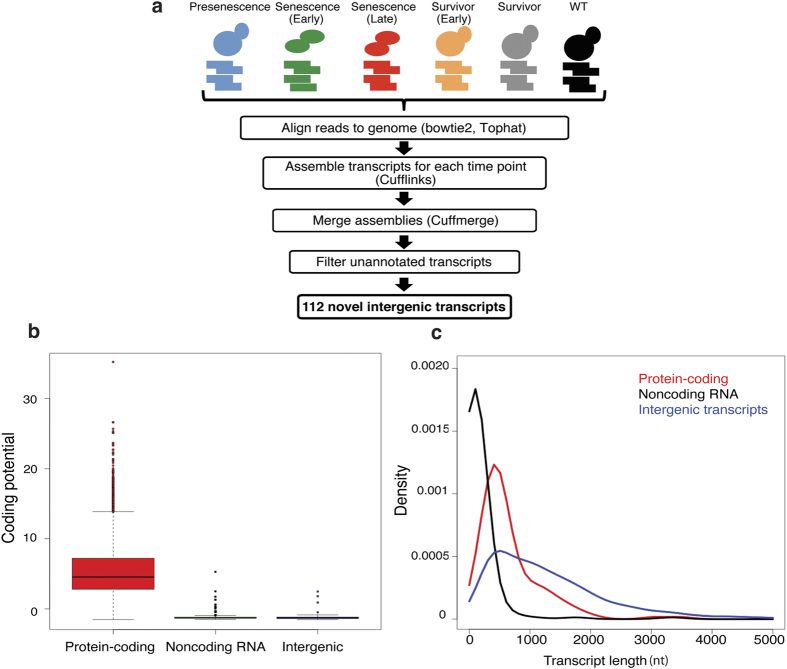
Characterization of novel intergenic transcripts. (**a**) Experimental workflow and analysis for analyzing RNA-seq data from *tlc1*∆ and nutrient-matched wild-type cells. (**b**) Intergenic transcripts show low coding potential. Comparison of the coding potential of known protein coding and noncoding genes with the intergenic transcripts calculated using CPC software. (**c**) Intergenic transcripts show a distinct length distribution. Length distribution of intergenic transcripts as well as known protein-coding and noncoding genes (P < 0.0001). Intergenic transcripts have a median length of 467 nts. Data points above 5000 nt are not shown. P value calculated using the two sample t-test.

RNA-seq reads from biological-replicate samples were mapped to the yeast genome and analyzed using the Tuxedo software suite^19^,^20^,^21^,^22^. Briefly, aligned reads underwent *de novo* assembly using Cufflinks prior to assembly into a consensus transcriptome using CuffMerge (see Materials and Methods). In total, 161 intergenic transcripts were identified and then compared with previously annotated genes; any transcripts that overlapped with known genes or annotated untranslated regions (UTRs) were removed (Fig. 2a). After this step, 112 potentially novel transcripts remained (Supp. Table 1). We also compared our results with previous transcriptome-profiling experiments and found that none of our candidates correspond to observed transcripts, although some were contained within larger unannotated transcripts^12^,^23^,^24^,^25^. It should be noted that many noncoding RNAs in yeast correspond to antisense transcripts that partially or completely overlap with known ORFs. Our analysis does not examine these transcripts and the lncRNAs reported are, therefore, a smaller, but distinct subset of the larger noncoding RNA landscape.

Widespread transcription of the yeast genome has been reported previously, with the majority of unannotated transcripts corresponding to several classes of unstable transcripts (XUTs, CUTs, NUTs)^14^. Often, the transcripts cannot be detected without depletion of either components of the exosome or stabilizing RNA-binding proteins. We did not observe any changes in Xrn1, Nrd1 or Rrp6 expression that could explain the novel transcripts in telomerase-negative cells (see below). Given that none of our candidates have been previously reported and they are not associated with any known pathways that increase transcript levels, they represent a potentially novel group of noncoding RNAs in *S. cerevisiae.* In addition, 41 of the noncoding RNAs are only expressed in telomerase-negative yeast and are therefore a novel class of transcripts, which we term TMLs (Telomerase-Mutant LncRNAs).

### Characterization of potential lncRNAs

To examine whether our candidates are likely to encode proteins, we used the Coding-Potential Calculator (CPC, version 0.9-r2)^26^ to measure the coding potential and ORF size in each transcript. The unannotated transcripts show similarly low coding potential to known noncoding RNAs in *S. cerevisiae* (Fig. 2b); significantly lower than that of protein-coding genes (P < 0.0001). This strongly suggests that the candidates do not encode a protein. Since lncRNAs are generally defined as transcripts greater than 200 nt, we also examined the length distribution of the intergenic transcripts. They are longer than annotated ncRNAs (median length = 467 nt) (Fig. 2c) and, therefore, we conclude that the candidates represent a group of novel lncRNAs.

**Figure 3:**
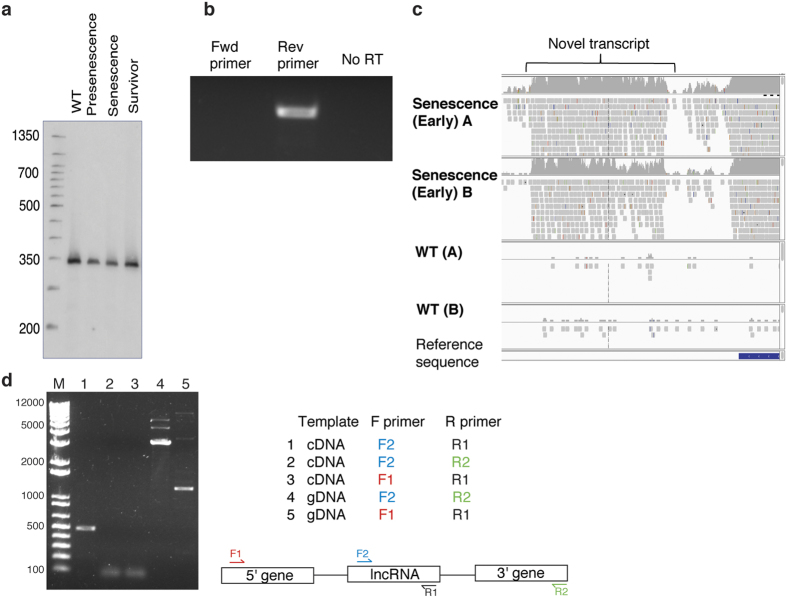
Validation of lncRNA candidates. (**a**) Tested candidates appear as discrete transcripts. Northern-blot analysis of one lncRNA candidate. Transcript appears as a distinct band and therefore likely has well-defined boundaries. (**b**) All tested candidates can be detected by RT-PCR. RT-PCR analysis of one candidate. (**c**) Mapped RNA-seq reads for one lncRNA candidate. Reads shown were visualized with Integrative Genomics Viewer software. (**d**) Tested candidates are not extensions of neighboring genes. RT-PCR was performed using lncRNA-specific primers or combining these with primers specific to neighboring genes (3′ or 5′ gene). As a control for PCR efficiency, PCR was also performed using the gene-specific and internal primers with genomic DNA (gDNA) as a template. RT-PCR using lncRNA primers along with primers in neighboring genes does not result in a product (lanes 2 and 3). **(a–c)** correspond to different transcripts. **(b,d)** represent the same transcript.

To determine whether the candidates represented distinct transcripts rather than extensions of nearby genes, we selected a subset of the most highly expressed lncRNAs to examine by northern blot analysis (Fig. 3). A single band, representing what we believe to be a single transcript, of length equivalent to that determined on the IGV viewer was present in all samples. This lncRNA did appear as an independent transcript and therefore does not represent extended transcription from neighboring genes. The expression of an additional 10 candidates was validated using RT-PCR (Fig. 3b, Supp. Table 1). We examined 5 of these in more detail by RT-PCR and determined that they do not represent extensions of adjacent gene transcripts (Fig. 3d, Supp. Table 1).

### Altered expression of novel lncRNAs in telomerase-negative cells

**Figure 4:**
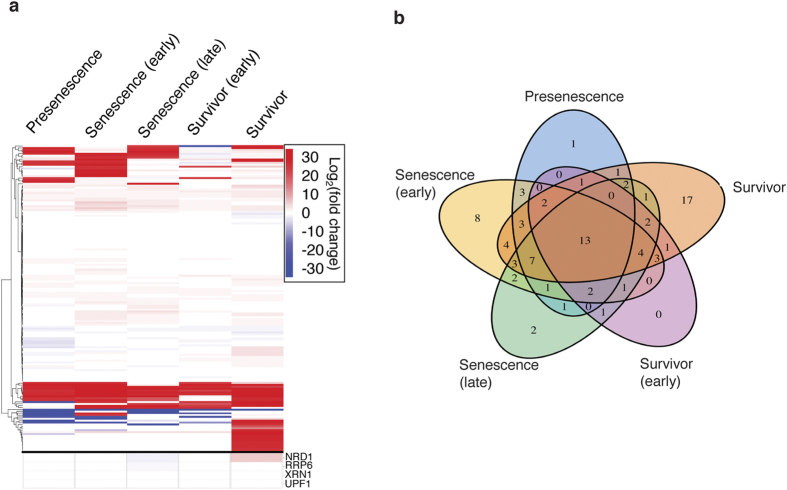
Many lncRNAs are differentially expressed in telomerase-negative cells. (**a**) Heatmap analysis of lncRNA candidate expression. Most lncRNAs are upregulated by two-fold or more (red). Any color (red or blue) indicates differential expression of at least two-fold. Differential expression of commonly mutated exosome and RNA-binding proteins is shown below black line. Differential expression is calculated by log_2_(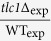) (**b**) Comparison of upregulated lncRNAs at each time point. Most lncRNAs (58/86) are upregulated in more than one time point.

We first examined the expression pattern of the lncRNAs in *tlc1*∆ cells relative to wild type. Over half of the novel lncRNAs are differentially expressed by two-fold or more in at least one of the time points tested (Fig. 4a). The majority of the differentially expressed lncRNAs were upregulated relative to wild type and in many cases the transcripts were not even detectable in wild-type cells. Thus, many of the lncRNAs are specific to telomerase-negative cells, providing an explanation as to why they have not been identified as lncRNAs previously.

Next, to identify any senescence or stage-specific lncRNAs, we compared the upregulated lncRNAs from each time point (Fig. 4b). However, what we found is that most lncRNAs (58/86) are upregulated in more than one time point. This suggests lncRNA transcription may be a more general response to telomerase deletion rather than a stage-specific response. In fact, only 12 transcripts were found exclusively in the senescence time points. Interestingly, however, survivor cells express the most stage-specific lncRNAs (17), comparable with the number expressed in every time point (13).

However, the expression level of the lncRNAs was generally quite low, with a median value of 19.9 FPKM (fragments per kilobase of transcript per million mapped reads), so despite the strong fold-change between the telomerase-negative and wild-type cells for many of the lncRNAs, only a small subset (between 5 and 22, depending on the time point) can be considered significantly differentially expressed without further analysis.

### A subset of lncRNAs show correlated expression with neighboring genes

**Figure 5:**
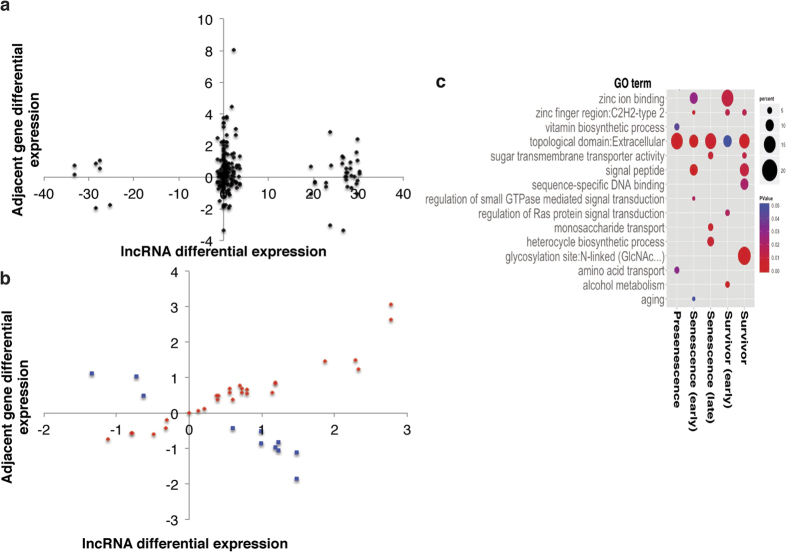
The lncRNA candidates may regulate expression of nearby genes. (**a**) Highly differentially expressed candidates do not show correlated expression with neighboring genes. 2D plot showing differential expression of lncRNAs and each adjacent gene. Late senescence time point is shown. Candidates fall into two groups (1) low differential expression, correlated with one or more adjacent gene and (2) high differential expression, no correlation with adjacent genes. (**b**) Correlated expression between a subset of lncRNAs and adjacent genes. Differential expression of lncRNAs showing either positive (red, R^2^ = 0.91) or negative (blue, R^2^ = 0.92) correlation with an adjacent gene plotted against differential expression of the adjacent gene. Differential expression is calculated by log_2_(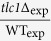) **(c)** Correlated genes are enriched with extracellular proteins. Functional enrichment analysis performed using DAVID and plotted using qplot in the ggplot2^44^ package in R studio. Proteins with an extracellular domain are enriched at each time point.

Since lncRNAs can regulate the expression of neighboring genes^27^, we examined correlation of each lncRNA differential expression with that of its adjacent genes at each time point (Fig. 5a). We define correlation as a linear relationship between the expression of a lncRNA and a neighboring gene for at least one time point. For most time points, approximately one-third of the lncRNAs show strong correlation (R^2^ 0.79–0.99) with at least one neighboring gene while, in the late-senescence samples, over half are correlated (shown in Fig. 5b; other time points in Supp. Fig. 3). Interestingly, the lncRNAs showing the largest differential expression relative to wild type show almost no correlation with either adjacent gene (R^2^ < 0.015, Fig. 5a).

Of the correlated lncRNAs, we observe more positive relationships than negative, indicating expression of the lncRNAs may promote expression of the neighboring gene. It is also possible that the correlation is the result of gene-transcript extensions being misidentified as lncRNAs, but because of the low expression levels it is difficult to distinguish between these possibilities.

We also tested if the correlated lncRNAs could be regulating genes with related functions. We analyzed the gene list using the Database for Annotation, Visualization and Integrated Discovery (DAVID)^28^ functional annotation tool. We find a strong enrichment for extracellular and membrane proteins (Fig. 5c). These groups are also enriched among the differentially expressed genes more broadly (data not shown) in every time point, suggesting lncRNAs may be contributing to the coordinated changes in expression of membrane and extracellular proteins in telomerase-negative cells.

**Figure 6:**
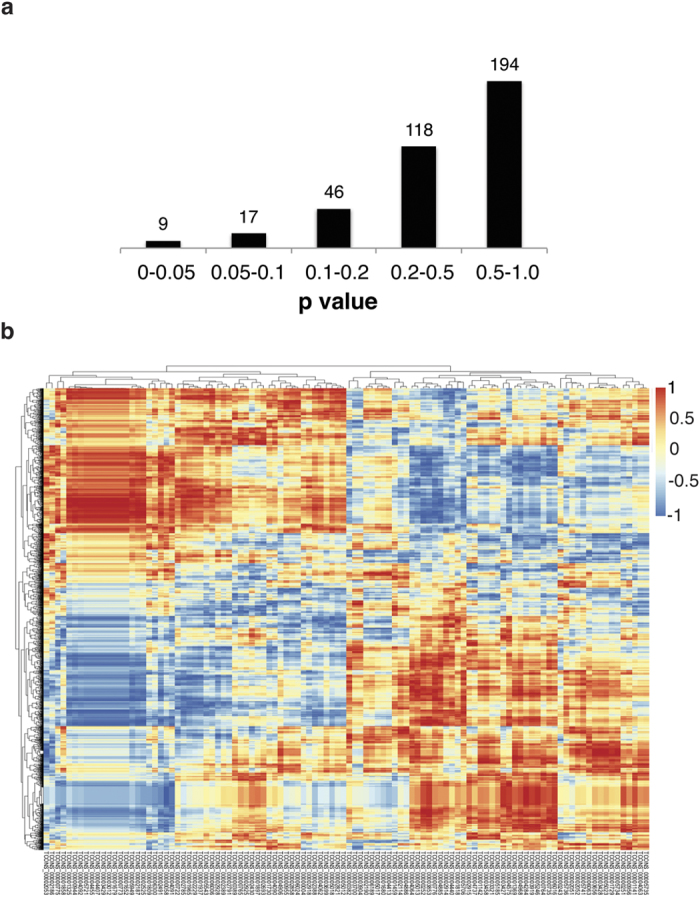
Correlated expression across time points. (**a**) 9 lncRNAs show significant Pearson correlation coefficients with an adjacent gene. Number of lncRNAs with the corresponding p-values for correlation with an adjacent gene. Most lncRNAs do not show significant similarity in expression with a neighboring gene throughout the time course. This may be partially due to the low expression levels of the lncRNAs. (**b**) Many lncRNAs show correlated expression with distant genes. Pearson correlation coefficients were calculated between the lncRNAs and the genome. Hierarchical clustering was performed prior to plotting to group genes showing similar patterns. LncRNAs are labeled below using unique identifiers. Perfect correlation would correspond to a coefficient of 1.0 while perfect anticorrelation would result in –1.0. Pairs showing high correlation or anticorrelation may belong to a co-regulated group of genes or represent *in trans* regulators. Full scores with gene names shown in Supp. Table 3.

Finally, we took a more stringent approach to verify the correlated lncRNAs and calculated the Pearson correlation coefficient between each lncRNA and neighboring genes. The low expression levels of the lncRNAs makes it difficult to identify statistically significant correlations in expression across all five time points, but we did find 9 lncRNAs showing potentially significant correlations (p < 0.05) (Summarized in Fig. 6a, Supp. Table 2). Of the 9 lncRNAs, 6 overlap with our previous analysis and represent the group most likely to be cis-regulators of neighboring genes. Many of the remaining lncRNAs show strongly correlated expression with non-adjacent genes (Fig. 6b, Supp. Table 3) and may modulate gene expression *in trans* or they may be co-regulated.

## Discussion

The close association of lncRNAs with both cancer and aging suggests they may also be involved in the cellular response to telomerase deletion and subsequent changes in telomere length. We have used RNA-seq to identify 112 putative novel lncRNAs in telomerase-negative yeast. The transcripts are unlikely to encode proteins and have a median length of 467 nt, which is well above the broadly accepted 200-nt threshold for classification as a lncRNA. Most of the lncRNAs were upregulated in at least one time point in the telomerase-negative cells and, in fact, many were undetectable in wild-type cells, which may explain why they have not been observed previously. Previous approaches to identify novel lncRNAs have generally utilized RNA-degradation mutants to stabilize transcripts^25^,^29^,^30^. However, our work reveals that, despite extensive study, there are undiscovered lncRNAs in *S. cerevisiae* that may become expressed as part of cellular responses to specific conditions, such as loss of telomerase and dysfunctional telomeres.

It is currently unclear what the functional roles of these lncRNAs are or even if these RNA molecules themselves are important, as opposed to transcriptional activity at their gene locations. Transcription of the ncRNAs SRG1 and IRT1 is sufficient to mediate repression of nearby genes via an interference mechanism^31^. Alternatively, a noncoding RNA generated from the telomeric repeats, TERRA, has been shown to interact with the yeast telomerase RNA, and may facilitate recruitment of telomerase to critically short telomeres^32^. Expression of some of these known lncRNAs may also be important for senescence, and this will be explored in future work.

Given the number of novel transcripts identified here, we believe it is most likely that the lncRNAs are modulating the expression of genes involved in the adaptive response to telomerase deletion. Indeed, several of the known lncRNAs in yeast, such as IRT1 and PWR1, are involved in transcriptionally regulating specific cell states such as sporulation and filamentous growth (reviewed in 45). We find many of the lncRNAs that we have identified are strongly correlated with the expression of adjacent genes, suggesting they act as cis-regulatory elements. Further testing will be required to fully elucidate the exact mechanism of action.

The genes showing correlated expression with a neighboring lncRNA are strongly enriched for both extracellular and membrane proteins (Fig. 5c). It has previously been reported that telomerase-negative cells undergo mitochondrial proliferation^10^,^33^ and may require changes in expression of membrane proteins as a result. Also, approximately half of the membrane- and extracellular-protein genes are near the telomeres. Silencing at the subtelomere decreases as telomeres shorten^34^,^35^, suggesting a potential link between telomere length and expression of novel lncRNAs from the subtelomeric DNA. Since these lncRNAs also show correlated expression with neighboring genes, it could indicate a programmed expression pathway that is initiated only when most telomeres are critically short, potentially mediated by Rap1 loss from subtelomeres during senescence^36^. Additionally, since these functional groups are enriched at every stage, this may represent an additional feature of the previously described telomerase-deletion response^10^. The correlated genes involved in cell-wall maintenance overlap somewhat with genes regulated by MUTs in meiotic cells^15^, which may indicate some similarities between the telomerase-deletion response and the meiotic program in yeast.

Some of the lncRNAs we observe at the subtelomere may overlap or behave similarly to those described previously in cells deficient for nonsense-mediated decay (NMD)^16^, referred to as Cytoplasmically Degraded Cryptic Unstable Transcripts (CD-CUTs). In particular, we see increased transcription 5′ of the zinc transporter, Zrt1. However we do not observe any changes in expression of the major components of the NMD pathway, suggesting the expression and stabilization of these transcripts may be the result of a parallel pathway in telomerase-negative cells. One possible explanation is that transcription at this locus is promoted in part by shortened telomeres. Cells lacking the central NMD component Upf1 do exhibit shortened telomeres in addition to NMD defects^37^,^38^.

Interestingly, the most differently expressed lncRNAs did not show any correlation with neighboring genes that might be expected either due to cis-regulation or spurious transcription. Thus, this group represents the lncRNAs most likely to act functionally as RNAs with targets elsewhere in the cell. Further studies will be required to determine the function of this relatively small group of lncRNAs (<20 transcripts), potentially beginning with localization studies to determine whether they remain in the nucleus or are exported to the cytoplasm.

Our analysis adds to the growing body of evidence that pervasive transcription of ncRNAs in yeast is an important component in the ability of the cell to adapt to both internal and external changes. In this case, telomerase deletion initiates a series of adaptive changes from cell-cycle arrest to initiation of alternative methods of telomere lengthening and we observe changes in lncRNA expression at each of these stages.

## Methods

### Growth and Characterization of yeast strains

The *TLC1*/*tlc1*∆:kan^r^ heterozygote^39^ was sporulated and dissected to generate the wild-type and *tlc1*Δ strains (*MAT*a *his3*Δ*1/his3*Δ*1 leu2*Δ*0/leu2*Δ*0 LYS2 MET15 ura3*Δ*0/ura3*Δ*0).* Genotyping was confirmed by replica-plating colonies onto appropriate media. Cells were passaged every 12 hours in YPAD media to maintain constant logarithmic growth. Small aliquots were removed for bud indexing and plating for colony formation. The remaining cells were harvested by vacuum filtration and the filters subsequently frozen in liquid nitrogen and then stored at –80 **°**C.

### RNA isolation

RNA isolation was carried out using hot-phenol method, as previously described^40^.

### Library preparation and RNA sequencing

RNA-seq libraries were prepared according to the protocol suggested by the TruSeq sample-prep guide by Illumina. Briefly, 3 to 5 μg of total RNA were used as input and treated with RiboZero to reduce ribosomal RNA. The remaining RNA was used for the construction of both strands of cDNA. Utilizing reagents form the Illumina kit, the ends of the cDNA fragments were repaired and a single A was ended to the 3′ termini. Illumina TruSeq adaptors were ligated to the ends of the DNA fragments and amplified. The library was then sequenced utilizing standard Illumina protocols and reagents on MiSeq instrumentation. Fastq files were generated by the software on the MiSeq, or by CASAVA 1.8 offline. Only reads that passed the Illumina chastity filter were used. Additional quality control analysis was performed using FastQC^41^. The mean per-base sequence quality for all samples was greater than 32.

### Data analysis

Reads were aligned to the UCSC SacCer3 genome (April 2011) using Tophat^20^ with the following parameters “-G SacCer3_SGD.gtf -I 2000 -i 40 --no-coverage-search UCSC/sacCer3/Bowtie2Index/genome”. Transcript boundaries and quantification were determined using cufflinks^20^,^21^,^22^. Data visualization was carried out in R Studio (http://www.R-project.org/)^42^.

Transcripts were considered novel if they (1) did not overlap with annotated transcripts or UTRs and (2) if the ends were more than 50-bp different from previously reported noncoding RNAs (SUTs, etc.). Telomerase Mutant LncRNAs (TMLs) correspond to lncRNAs that are upregulated relative to wild type in at least one time point while never being downregulated relative to wild type in any time point.

### Telomeric Southern blotting

Southern blotting was used to evaluate telomere length, as previously described^43^.

## Additional Information

**How to cite this article**: Niederer, R. O. *et al.* Identification of novel noncoding transcripts in telomerase-negative yeast using RNA-seq. *Sci. Rep.* **6**, 19376; doi: 10.1038/srep19376 (2016).

## Supplementary Material

Supplementary Information

Supplementary Table 1

Supplementary Table 2

Supplementary Table 3
