# Supplementary material for: Identification of novel noncoding transcripts in telomerase-negative yeast using RNA-seq
Source: Sci Rep. 2016 Jan 20;6:19376. doi: 10.1038/srep19376 (PMC4726298; doi:10.1038/srep19376)
Supplement: Supplementary Information [file srep19376-s1.pdf]

Supplementary Information for

Identification of novel noncoding transcripts in telomerase-negative yeast using RNA-seq

Rachel O. Niederer[1], Nickolas Papadopoulos[2], and David C. Zappulla[1*]

[1] Department of Biology, Johns Hopkins University, Baltimore, MD, 21218

[2] Ludwig Center for Cancer Genetics and Therapeutics, The Johns Hopkins Sidney Kimmel Comprehensive Cancer Center, Baltimore, MD 21231 USA

[*]Corresponding author. Phone: (410) 516-8749. Email: zappulla@jhu.edu

Supplemental Figure-1 (Zappulla)

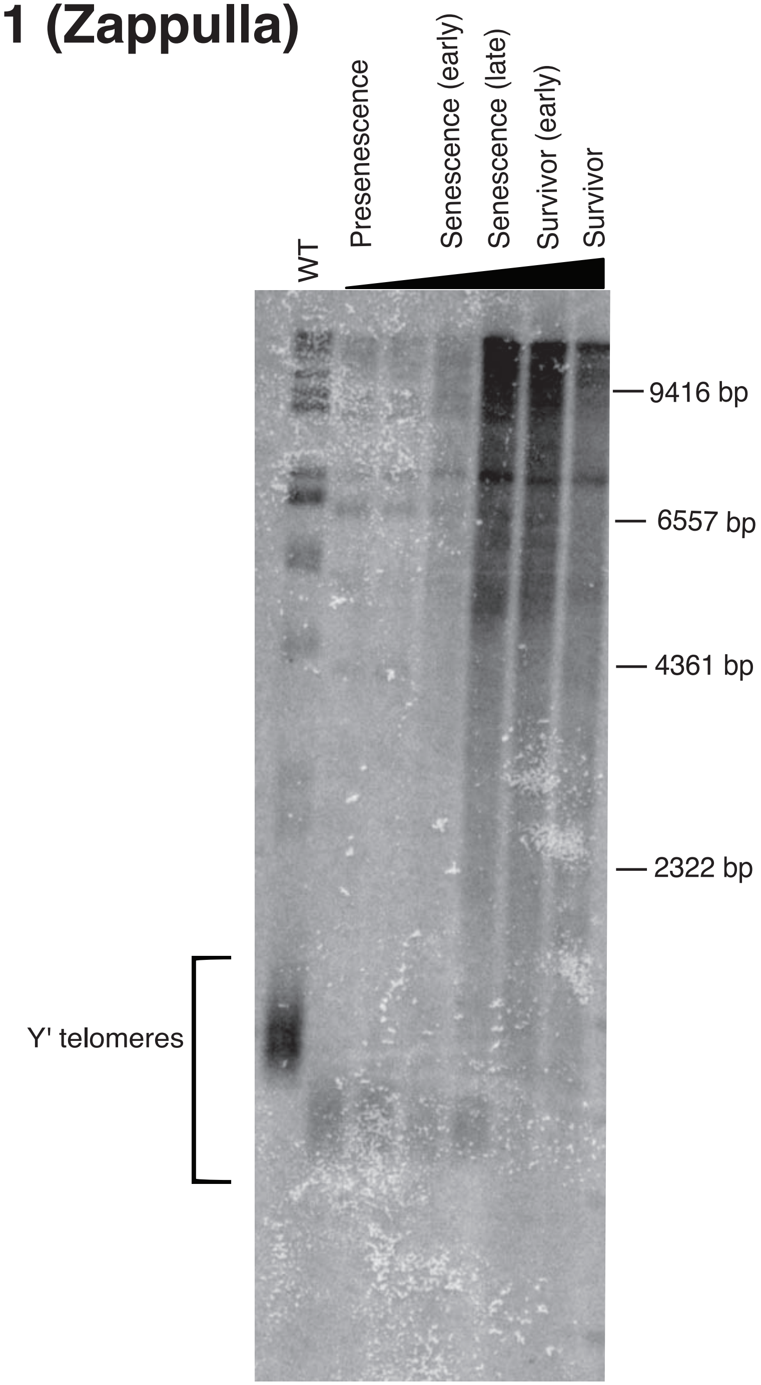

Supplemental Figure 1. Telomere Southern blot. Representative telomere Southern blot of *tlc1Δ* cells. Increasing time is indicated by a black triangle. Samples are labeled according to the categorized time point used for sequencing.

Supplemental Figure-2 (Zappulla)

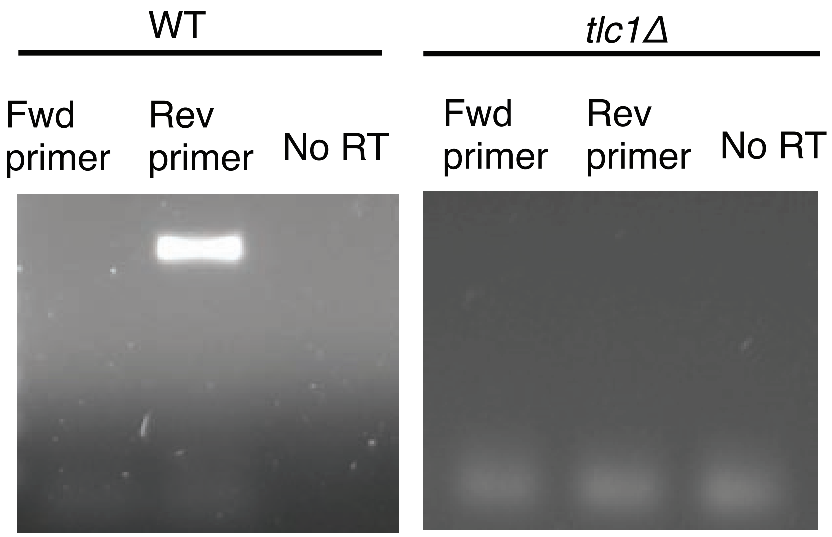

Supplemental Figure 2. RT-PCR verification of *tlc1Δ* cells. RT-PCR detection of TLC1 transcript in WT and *tlc1Δ* cells. As expected, the transcript is only detectable using a reverse primer in WT cells. All tested lncRNA candidates summarized in supplemental table 1.

Supplemental Figure-3 (Zappulla)

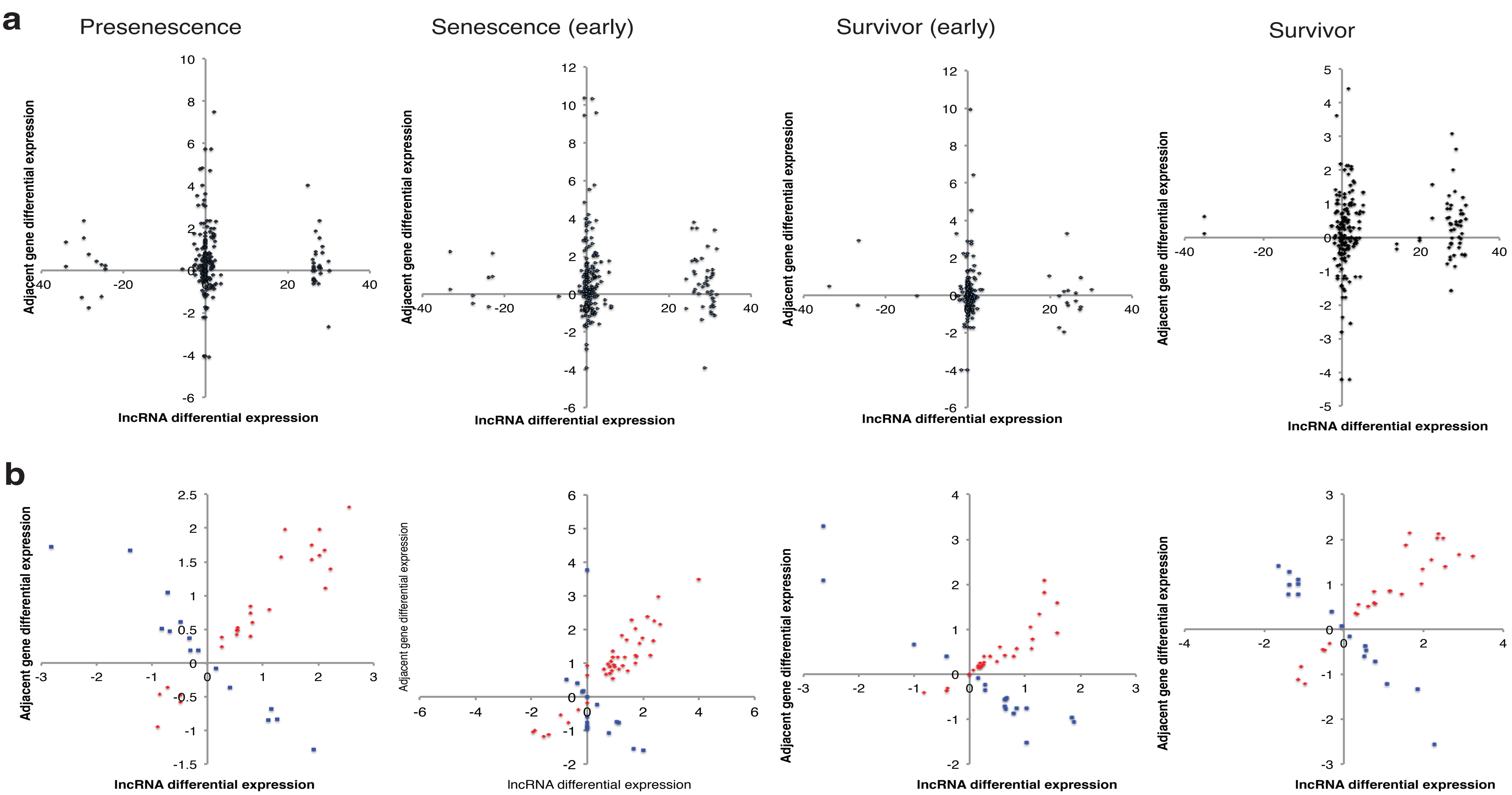

c

	Presenescence	Senescece (early)	Senescece (late)	Survivor (early)	Survivor
negatively correlated	-31	4	-50	-18	-22
postively correlated	38	11	55	38	50

Supplemental Figure 3. Correlation analysis of lncRNAs and adjacent genes. (a) Differential expression of lncRNAs plotted against differential expression of adjacent genes. (b) Subset of lncRNAs and corresponding adjacent genes showing correlated expression at each timepoint. Negatively correlated pairs shown in blue, positively correlated pairs shown in red. **(c) Percent difference in the number of observed vs expected correlated genes (P <0.001)**. Expected values calculated using a randomly generated dataset . P value calculated using Chi-squared test.
